# Non-medical use of prescription drugs by young men: impact of potentially traumatic events and of social-environmental stressors

**DOI:** 10.1080/20008198.2018.1468706

**Published:** 2018-05-09

**Authors:** Ansgar Rougemont-Bücking, Véronique S. Grazioli, Simon Marmet, Jean-Bernard Daeppen, Mélissa Lemoine, Gerhard Gmel, Joseph Studer

**Affiliations:** a Alcohol Treatment Center, Centre Hospitalier Universitaire Vaudois CHUV, Lausanne, Switzerland; b Chair of Psychiatry and Psychotherapy, University of Fribourg, Department of Neurosciences and Movement Science (NMS), Psychiatry and Psychotherapy, Fribourg, Switzerland; c Addiction Suisse, Lausanne, Switzerland; d Centre for Addiction and Mental Health, Toronto, Canada; e Frenchay Campus, University of the West of England, Bristol, UK

**Keywords:** Non-medical use of prescription drugs, traumatic stress, family functioning, peer influence, parental monitoring, neglect, sexual assault, physical assault, uso no médico de fármacos recetados, estrés traumático, funcionamiento familiar, influencia de los compañeros, control parental, negligencia, agresión sexual, agresión física, 非医疗用处方药, 创伤性应激, 家庭功能, 同伴影响, 家长监督, 忽略, 性侵犯, 身体攻击, • This study tested the associations between various forms of stress on non-medical use of prescription drugs (NMUPD) by young men in Switzerland.• NMUPD was highly associated with the number of potentially traumatic events young men were confronted with earlier in life, as well as with having recently had problems with family or friends.• Measures describing family functioning, such as a lack of parental support or monitoring, showed almost no associations with NMUPD.• Physical and sexual assaults showed more associations with NMUPD when perpetrated by strangers than when perpetrated by acquaintances.

## Abstract

**Background**: Non-medical use of prescription drugs (NMUPD) is an increasing phenomenon associated with physical and psychological consequences. This study investigated the effects of distinct forms of stress on NMUPD.

**Methods**: Data from 5308 young adult men from the Swiss cohort study on substance use risk factors (C-SURF) were analysed regarding NMUPD of sleeping pills, tranquilizers, opioid analgesics, psychostimulants, and antidepressants. Various forms of stress (discrete, potentially traumatic events, recent and long-lasting social-environmental stressors) during the period preceding the NMUPD assessment were measured. Backward log-binomial regression was performed and risk ratios (RR) were calculated.

**Results**: NMUPD was significantly associated with the cumulative number of potentially traumatic events (e.g. for opioid analgesics, RR = 1.92, *p* < .001), with problems within the family (e.g. for sleeping pills, RR = 2.45, *p* < .001), and the peer group (e.g. for tranquilizer use, RR = 2.34, *p* < .01). Factors describing family functioning in childhood showed very few significant associations. Sexual assault by acquaintances was associated only with use of sleeping pills (RR = 2.91, *p* p <.01); physical assault by acquaintances was not associated with NMUPD. Physical (e.g. for psychostimulants, RR = 2.01, *p* < .001) or sexual assaults (e.g. for antidepressants, RR = 4.64, *p* < .001) perpetrated outside the family context did show associations with several drug categories.

**Conclusion**: NMUPD appears to be more consistently associated with discrete and potentially traumatic events and with recent social-environmental stressors than with long-lasting stressors due to family functioning during childhood and youth. Physical and sexual assaults perpetrated by strangers showed more associations with NMUPD than those perpetrated by a family member.

## Abstract (es)

**Introducción**: El uso no médico de fármacos recetados (UNMFR) es un fenómeno creciente asociado con consecuencias físicas y psicológicas. Este estudio investigó los efectos de distintas formas de estrés en el UNMFR.

**Métodos**: Se analizaron los datos de 5,308 varones adultos jóvenes del estudio suizo de cohorte sobre los factores de riesgo del uso de sustancias (C-SURF) con respecto a UNMFR de pastillas para dormir, tranquilizantes, analgésicos opioides, psicoestimulantes y antidepresivos. Se midieron diversas formas de estrés (eventos discretos potencialmente traumáticos, estresores socioambientales recientes y duraderos) durante el período anterior a la evaluación del UNMFR. Se realizó una regresión logística- binaria hacia atrás y se calculó el riesgo relativo (RR).

**Resultados**: UNMFR se asoció significativamente con el número acumulativo de eventos potencialmente traumáticos (e.g. para analgésicos opioides, RR = 1.92, *p* <.001), con problemas dentro de la familia (e.g. para pastillas para dormir RR = 2.45, *p* <.001). y el grupo de iguales (e.g. para el uso de tranquilizantes RR = 2.34, *p* <.01). Los factores que describen el funcionamiento familiar en la infancia mostraron escasas relaciones significativas. La agresión sexual por conocidos solo se asoció con el uso de pastillas para dormir (RR = 2.91, *p* = <. 01) mientras que la agresión física por conocidos no se asoció con UNMFR. La actividad física (e.g. para psicoestimulantes RR = 2.01, *p* <.001) o agresiones sexuales (e.g, para antidepresivos RR = 4.64, *p* <.001) perpretados fuera del contexto familiar mostraron asociaciones con varias categorías de fármacos.

**Conclusión**: El UNMFR parece asociarse de forma más consistente con eventos discretos y potencialmente traumáticos y con estresores sociales y ambientales recientes que con factores estresantes de larga duración debidos al funcionamiento familiar durante la infancia y la juventud. Las agresiones físicas y sexuales perpetradas por extraños mostraron más asociaciones con el UNMFR que las cometidas por un miembro de la familia.

## Abstract (zh)

**背景：**处方药的非医疗用途（Non-Medical Use of Prescription Drugs, NMUPD）日益增加，与躯体和心理方面的一系列后果有密切联系。这项研究调查了不同形式的压力对NMUPD的影响。

**方法：**对来自瑞士的物质使用风险因素研究项目（C-SURF）的5308名年轻成年男性的数据进行了分析，所使用药物涉及安眠药、安定药、阿片类镇痛药、精神兴奋药和抗抑郁药的NMUPD。对NMUPD评估前各种形式的压力（分散的，潜在的创伤性事件，最近的和长期的社会环境压力源）进行测量。使用反向对数二项回归（Backward log-binomial regression）进行分析，并计算对应风险比。

**结果：**NMUPD与潜在创伤性事件的累积数量（例如阿片类镇痛药，RR = 1.92，*p* <.001），与家庭中存在的问题（例如安眠药RR = 2.45，*p* <.001），和同伴组（例如，镇静剂使用RR = 2.34，*p* <0.01）呈显著相关。与童年期家庭功能的极少数因素呈现显著相关。来自熟人的性侵犯只与使用安眠药有关（RR = 2.91，*p* = <.01），而来自熟人的躯体攻击与NMUPD无关。在家庭环境之外发生的的躯体攻击（例如对于精神兴奋剂RR = 2.01，*p* <.001）或性侵犯（例如对于抗抑郁药RR = 4.64，*p* <.001）显示与某几类药物使用有关。

**结论：**与儿童和青年期的家庭功能失衡所带来的长期压力源的作用相比，NMUPD似乎与分散的、潜在的创伤事件，以及近期遭遇的社会环境方面的应激源更为稳定的相关。躯体和性方面的侵犯与NMUPD的关系中，来自陌生人的侵犯比来自家人的侵犯与NMUPD相关更高。

## 1. Introduction

Prescribed medications are essential for the treatment of anxiety, insomnia, depression, and physical pain which are symptoms attributable to psychiatric or somatic diseases (Wigman et al., 2013). However, the high prevalence of unpleasant symptoms, such as those mentioned above, and the high availability of prescription drugs in the general population, explain why certain medications are taken outside of the well-defined medical setting. Several studies have shown that the non-medical use of prescription drugs (NMUPD) has become the second most common form of ‘illicit’ substance use (SU), after cannabis use (Baggio, Studer, Mohler-Kuo, Daeppen, & Gmel, 2014; Ford & Arrastia, 2008). NMUPD is defined as the use of a prescription drug either without a physician’s prescription, for a longer duration than prescribed, for reasons other than those for which it was intended, or in higher doses than prescribed (Barrett, Meisner, & Stewart, 2008).

The known health risks of NMUPD are due mainly to the effects of acute intoxication or to long-term effects such as the development of an addiction or the worsening of an undiagnosed and untreated medical condition (Ali et al., 2015; Baggio et al., 2014). These potential harms put prescribing physicians and the regulatory authorities under a lot of pressure to control and possibly limit the amount of prescriptions.

There is consistent evidence that NMUPD is a means of self-medication to dampen the physical and psychological pain arising from stressful life events (Brands, Paglia-Boak, Sproule, Leslie, & Adlaf, 2010; Johnston, 2009; Pinch, Heck, & Vinal, 1986; Zellner, Watt, Solms, & Panksepp, 2011). Whereas consistent similarities in the perception of physical and psychological pain as well as the ability of pain medication to diminish not only physical but also psychological suffering to some extent have been demonstrated (Eisenberger, 2012), studies of NMUPD commonly focus on medications that have psychotropic properties.

Various classes of medication are used according to specific psychosocial factors, such as ethnicity, gender, educational and social status, as well as to the specific expectations individuals have about these substances (Boyd, McCabe, Cranford, & Young, 2006; Conn & Marks, 2014; Kelly et al., 2013; Rigg & Ibanez, 2010). For instance, opioid pain medication was used by adolescents to ‘relieve pain’, rather than to ‘get high’ (McCabe, West, & Boyd, 2013). These different preferences have also been shown to evolve distinctly for each medication over time, further reinforcing the idea that they are chosen with specific regard to a perceived individual problem and the precise effects that the substance can have on it (McCabe, Kloska, Veliz, Jager, & Schulenberg, 2016). In addition, it is well established that NMUPD by young people often shows concomitant use of alcohol or illicit substances (Boyd, Esteban McCabe, & Teter, 2006; Ford, 2009; Kelly, Wells, Pawson, LeClair, & Parsons, 2014).

As specific substance classes of prescription medication can be taken either solely or concomitantly with other classes of prescription medication or with illicit substances, methodological difficulties arise when investigating the relationships between psychosocial factors and NMUPD or SU. Also, although NMUPD is an umbrella term for many distinct classes of drugs with different pharmacological properties, many studies focus on single substance classes, such as stimulants or opioids (Herman-Stahl, Krebs, Kroutil, & Heller, 2006; Joffe, 2014; McCabe et al., 2013; Sung, Richter, Vaughan, Johnson, & Thom, 2005). Further, specific NMUPD depends also on personality traits: whereas sociability was inversely related to NMPDU of sleeping pills and tranquilizers, it was shown that sensation seeking was solely related to use of psychostimulants (N’Goran et al., 2015). These findings suggest that a narrow scope of investigation focusing on the distinct classes of NMUPD better takes into account the specific effects and availabilities of the substances on the one hand, and the needs and previous experience of their users on the other.

Existing literature has revealed that major stressful life events are associated with NMUPD. A history of witnessing violence, experiencing a natural disaster, or a lifetime history of Post-Traumatic Stress Disorder (PTSD) were associated with an increased likelihood of NMUPD (Lowe, Sampson, Young, & Galea, 2017; McCauley et al., 2010; Quinn et al., 2016), particularly with the increased non-medical use (NMU) of opiates (Smith, Smith, Cercone, McKee, & Homish, 2016).

Therefore, main stressors investigated in the present study were reports of physical violence or of other discrete and possibly traumatic situations, such as accidents or severe illness. A special focus was whether a history of physical or sexual assault gave a different outcome with regard to NMUPD if assaults took place within or outside the family setting. We hypothesized that individuals with a history of physical or sexual assault show higher rates of NMUPD than those without such histories, with rates even higher when the assault was perpetrated by acquaintances in comparison to strangers. The rationale to explain this hypothesis lies in the fact that the destructive effects of interpersonal violence is even more pronounced if the maltreatment is perpetrated by persons who are expected to act in a respectful and loving manner, such as parents, family members, or care-givers (Bureau, Jodi, & Lyons-Ruth, 2010; Charuvastra & Cloitre, 2008; Pedersen, 2004).

Many highly traumatic events occur typically at single occasions with a clearly defined beginning and ending. However, stress may not only stem from distinct traumatic events but also from chronic daily-life hassles within the social-environmental setting in which an adolescent is growing up. These repetitive minor events may be even more predictive of stress-related impairments than single major life events (Chamberlain & Zika, 1990; McIntyre, Korn, & Matsuo, 2008). Such hassles may occur in different contexts, whereby family and peer surroundings are two of the most important ones for adolescents (Booth & Anthony, 2015). As to the influence of family factors, it has been shown that adolescents were less likely to engage in NMUPD when they had strong, positive bonds with their family or school environments (Ford, 2009). It was also shown that adolescents with a respectful attitude towards their parents were at decreased risk to practice NMUPD, whereas SU by parents was identified as a risk factor for NMUPD by their children (Tucker et al., 2015). With regard to peer influence, factors such as peers offering substances and peer approval of SU were also shown to be associated with increased NMUPD (Tucker et al., 2015). Other factors, such as perceptions of peers’ SU (D’Amico & McCarthy, 2006) or peers’ attitudes to SU, have also been established to influence NMUPD in adolescents (Ford, 2008b; Peralta & Steele, 2010).

Thus, in addition to the potentially traumatic, discrete events, the present study also looks at rather chronic and more subtle sources of stress within the social environment, namely severe problems with peers or family, an unsatisfactory relationship with parents, a history of a severe mental health disorder in a parent, and a lack of parental support or monitoring.

In sum, the stressors investigated can be conceptualized in the following way. On the one side, highly stressful, discrete, and potentially traumatic events were assessed. In the cases of physical and sexual aggression, a distinction of whether the aggression was perpetrated by a family member or by a stranger was made. On the other side, long-lasting social-environmental stressors which are not necessarily of traumatic nature were investigated. These stressors are known to be relevant risk factors for developing substance use disorders or NMUPD (Sinha, 2008). In addition to investigating NMPDU as an umbrella behaviour, it is also investigated whether potentially traumatic events and long-lasting environmental stressors are differentially associated with distinct drug classes.

The present study includes only young men. The literature concerning gender effects on NMUPD is sparse and inconsistent. Women appear to be at a higher risk to practice NMUPD, and there is evidence that social stress contributes to increased NMUPD in women (Ford, Reckdenwald, & Marquardt, 2014). Also, there are gender differences concerning motivation. Young women tend to use prescribed medications for reasons other than initially prescribed, whereas young men tend to ‘illicitly’ access and to use medication which was not prescribed for them (Peralta, Stewart, Steele, & Wagner, 2016; Simoni-Wastila, Ritter, & Strickler, 2004). Male gender, but more particularly masculine attitudes, such as risk taking or the lack of acknowledgement of the own psychic suffering (‘toughness’) have been shown to be associated with NMUPD (Ford, 2008a; Peralta et al., 2016), making young men a particular target for studies on NMUPD. However, despite this ‘masculinity risk factor’ for developing NMUPD there are no studies that focus on stress effects in male populations with regard to NMUPD. Thus, this study aimed to investigate the effects of specific forms of stress during adolescence on NMUPD in young men.

## 2. Methods

### 2.1. Study design

Data were drawn from the Swiss Cohort Study on Substance Use Risk Factors (C-SURF), focussing on risk and protective factors of SU and other behaviours of young Swiss men over time. The study protocol was approved by the Human Research Ethics Committee of the Canton Vaud (protocol number 15/07).

Participants were enrolled at three of Switzerland’s six recruitment centres that conscript men for military service; these cover 21 of the country’s 26 cantons. Attending military recruitment is mandatory for all Swiss men at around the age of 19. Enrolling participants at these locations thus provided C-SURF with a representative sample of young Swiss men. The centres were involved solely to enrol participants; however, the study was conducted independently of the military (questionnaires were sent to the home addresses) and regardless of the fact whether participants provided military or civilian service or obtained an exemption. The present study used C-SURF data collected from the initial baseline assessment of those enrolled (socio-demographic characteristics and social-environmental stressors) and from the follow-up evaluation (assessment of traumatic experiences and NMUPD), which took place about 15 months later.

### 2.2. Participants

An initial group of 7556 conscripts gave their written consent to participate. Of these, 5987 (79.2% of the initial group) participated in the baseline assessment, and 5479 (91.5%) completed the follow-up assessment 15 months later. One hundred and seventy-one participants were excluded from the analysis due to missing data. The final sample consisted of 5308 participants. More information on the enrolment procedure, non-consent, and non-response bias was provided in previous studies by our group (Gmel et al., 2015; Studer et al., 2013).

### 2.3. Outcome variables

#### 2.3.1. NMUPD

All participants were asked at follow-up whether they had taken prescription drugs for NMU during the past 12 months (‘used’ coded 1; ‘not used’ coded 0). Substances were: (1) sleeping pills, (2) tranquilizers, (3) opioid-based analgesics, (4) stimulants, or (5) antidepressants. For each substance group, several examples of specific pharmacological agents and their commonly commercialized product names were listed. Additionally, an any NMUPD group (use of at least one of the aforementioned prescription drugs) was constructed.

### 2.4. Independent variables (IV)

#### 2.4.1. Assessment of rather discrete, potentially traumatic events

Exposure to stressful potentially traumatic incidents (such as traffic accidents, earthquakes, severe illness, or injury) was assessed at the 15-month follow-up using 12 stressful events of the Post-traumatic Diagnostic Scale (PDS-enhanced; see Foa, Cashman, Jaycox, & Perry, 1997). This list was complemented with six events drawn from the Trauma History Questionnaire (THQ; see Hooper, Stockton, Krupnick, & Green, 2011) and two from the Life Event Checklist (Gray, Litz, Hsu, & Lombardo, 2004). Questionnaires are available on www.c-surf.ch (C-SURF, 2017). Participants were asked to indicate every event they had experienced during their life and in the last 12 months. Only events that had occurred more than 12 months prior to the follow-up assessment were included in the present analysis, thus ensuring that traumatic events preceded NMUPD. Four items from this list asking about physical or sexual assault perpetrated either by family members (or close acquaintances) or by strangers (unknown persons) were used as individual variables. The other 16 items were used to determine whether participants suffered none, one or two, or three or more potentially traumatic events in their life.

#### 2.4.2. Assessment of chronic, social-environmental stressors

As for potentially traumatic events, assessment of six social-environmental stressors focused on experiences that had occurred more than 12 months prior to measurement of NMUPD at follow-up: (1) Recent, past 12-month prevalence of serious problems with family members assessed at baseline (which means about 15 months prior to follow-up) was scored as no problems with family, one or two problems, and three or more problems; (2) Serious problems with friends were measured in the same way. Long-lasting social-environmental stressors were measured as follows (social-environmental stressors 3 to 6): (3) Perceived quality of participants’ relationships with their parents, before reaching the age of 18, were measured on a five-point Likert scale (1 = very satisfactory; 5 = very unsatisfactory relationship). The means of these responses were dichotomized at a cut-off of 3 (≥ 3 was coded as 1) thereby separating the responses into one group with satisfactory relationships (coded 0) and another with unsatisfactory relationships (coded 1). The perceived quality of parenting during childhood and youth was assessed by means of four questions. Two of these were related to whether there was a presence or absence of parental monitoring (*My parents knew where I spent my evenings, My parents knew with whom I spent my evenings*) and two assessed whether participants believed that they had been raised in an emotionally supportive family environment (*I received warmth and affection from my parents, My parents supported me*). Responses were given on a five-point Likert scale (1 = almost always; 5 = almost never). The means for each variable were dichotomized at a cut-off of 3 (≥ 3 was coded as 1) and recorded as a lack (coded 1) or presence (coded 0) of either parental monitoring (factor 4) or emotional support (factor 5). Questions for measures 1–5 stem from the European School Survey Project on Alcohol and Drugs (Hibell et al., 2012). Finally (factor 6), family history of a mental health disorder in a parent (including SU disorder) was assessed using the Addiction Severity Index (McLellan, Luborsky, Woody, & O’Brien, 1980). A parental mental illness was assigned (coded 1) when either the participant’s mother or father suffered from at least one mental health disorder.

### 2.5. Confounding socio-demographic variables

Age, perceived family income, and the participant’s highest level of educational attainment (number of years of training at school or university) were used to adjust for socio-demographic differences.

### 2.6. Statistical analysis

Data were analysed using version 23.0 of the SPSS. Unadjusted contingency tables and chi-square tests were used for bivariate associations. To avoid odds ratios as biased proxies of relative risk (RR) estimates, we used log-binomial regressions instead of logistic regressions for multiple adjusted models yielding RR (Taeger, Sun, & Straif, 1998; Williamson, Eliasziw, & Fick, 2013). Separate models were estimated for each group of NMUPD and for any use of NMPDU. To identify the most important predictors of NMUPD and to get parsimonious models due to some overlap between IV, a backward elimination approach (*p*_out_ ≥ .05) was used, starting with all IV and forcing confounding socio-demographics into the model. Before conducting regression models, multicollinearity was checked using the variance inflation factor (VIF) for each explanatory variable. No problem of multicollinearity was detected, as the highest VIF value (all VIFs < 1.30) was well below the thresholds (≥ 5 or ≥ 10) generally considered as evidence of multicollinearity (see O’brien, 2007).

## 3. Results

### 3.1. Sample description and bivariate associations in unadjusted model

The mean age of participants at follow-up was 21.3 years (standard deviation, 1.3 years). Prevalence of sexual assault by a family member and by a stranger was 1.2 and 1.0%, respectively (Table 1). Opioid analgesics had been used by as many as 6.1% of all participants, making them the most used NMUPD, and 9.4% of all participants used at least one NMUPD. Generally, about 70% of users at follow-up have initiated NMUPD between baseline and follow-up assessment (see Table 1). For example, of the 152 users of sleeping pills at follow-up (2.9% of the total sample), 107 (70.4% of all users at FU1) have initiated the use between baseline and follow-up assessment. The degree of overlap between the different NMUPD groups was moderate: out of the 500 participants reporting using at least one NMUPD, 357 (71.4%) reported using only one NMPD, as opposed to 143 (28.6%) participants using two or more NMUPD. Most of the bivariate associations were significant (56 out of 66), showing that all stressors were positively associated with at least one NMUPD class (Table 2).

**Table 1.** Descriptive statistics of sample.

	*N* (total = 5308)	%
Education		
9 years	394	7.4
12 years	2461	46.4
13 years or more	2453	46.2
Family income		
below average	744	14.1
average	2202	41.4
above average	2362	44.5
Physical assault by a family member (ref. no)	217	4.1
Sexual assault by a family member (ref. no)	64	1.2
Physical assault by a stranger (ref. no)	518	9.8
Sexual assault by a stranger (ref. no)	51	1.0
Number of potentially traumatic events (ref. none)		
one or two	1683	31.7
three or more	683	12.9
Problems with family within past year (ref. no)		
once or twice	877	16.5
three times or more	348	6.5
Problems with friends within past year (ref. no)		
once or twice	1065	20.1
three times or more	231	4.4
Unsatisfactory relationship with parents (ref. satisfied)	723	13.6
Lack of parental support (ref. adequate support)	472	8.9
Lack of parental monitoring (ref. adequate monitoring)	970	18.3
Parents having mental health problems (ref. no)	589	11.1
Sleeping pill use at FU1 (initiated at FU1)	152 (107)	2.9 (70.4)
Tranquilizer use at FU1 (initiated at FU1)	133 (97)	2.5 (72.9)
Opiate based analgesics use at FU1 (initiated at FU1)	323 (230)	6.1 (71.2)
Psychostimulant use at FU1 (initiated at FU1)	89 (57)	1.7 (64.0)
Antidepressant use at FU1 (initiated at FU1)	65 (51)	1.2 (78.5)
Any NMUPD	500 (318)	9.4 (63.6)

FU = follow-up assessment; NMUPD = non-medical use of prescription drugs; ref. = reference. Initiated gives *n* of those who used it first time at FU1, and the corresponding % is the percentage of all using it at FU1, who initiated it at FU1.

### 3.2. Log-binomial regression: associations between various stressors and distinct groups of NMUPD classes

Multiple, log-binomial regressions (Table 3) revealed that among the potentially traumatic events only the cumulative number of events was consistently significantly associated with all the NMUPD outcomes. Physical assault within the family setting was not associated with any of the outcomes (RR between 1.47 and 3.76). Other stressors showed distinct associations with drug classes. Sexual assault by a family member was only significantly associated with the NMU of sleeping pills (RR = 2.91, *p* < .01). Physical assault by a stranger was significantly associated with the NMU of psychostimulants (RR = 2.01, *p* < .001) and opioid analgesics (RR = 1.46, *p* < .01), whereas sexual assault by a stranger was significantly associated with the NMU of tranquilizers (RR = 3.01, *p* < .01) and antidepressants (RR = 4.64, *p* < .001).

**Table 2.** Covariate statistics of all evaluated variables towards all NMUPD outcomes.

	Sleeping pill use at FU			Tranquilizer use at FU			Opioid analgesic use at FU		
*N* = 5308	no (%)	yes (%)	*p*	no(%)	yes (%)	*p*	no (%)	yes(%)	*p*
Physical assault by family member (ref. no)	4.0	7.2	.047	4.0	7.5	.043	3.9	6.5	.024
Sexual assault by family member (ref. no)	1.1	5.9	< .001	1.1	6.0	< .001	1.1	3.1	.001
Physical assault by a stranger (ref. no)	9.7	13.2	.152	9.4	23.3	< .001	9.2	18.0	< .001
Sexual assault by a stranger (ref. no)	0.9	4.6	< .001	0.9	5.3	< .001	0.8	3.1	< .001
Number of potentially traumatic events (ref. none)			< .001			< .001			< .001
one or two	31.6	36.2		31.7	32.3		31.3	38.7	
three or more	12.5	24.3		12.4	29.3		12.3	22.3	
Problems with family within past year (ref. no)			< .001			< .001			< .001
once or twice	16.4	21.1		16.2	29.3		16.2	21.1	
three times or more	6.2	18.4		6.3	18.0		6.1	13.0	
Problems with friends within past year (ref. no)			< .001			< .001			< .001
once or twice	19.7	31.6		19.7	32.3		19.5	29.1	
three times or more	4.2	9.2		4.1	14.3		4.1	7.7	
Unsatisfactory relationship with parents (ref. satisfactory)	13.2	26.3	< .001	13.3	24.8	< .001	13.4	17.3	.044
Lack of parental support (ref. adequate support)	8.7	16.4	.001	8.7	16.5	.002	8.8	10.2	.388
Lack of parental monitoring (ref. adequate monitoring)	18.1	23.7	.080	18.2	21.1	.401	18.2	19.5	.555
Parents having mental health problems (ref. no)	10.8	21.7	< .001	10.8	24.1	< .001	11.1	13.3	.191
	Psychostimulant use at FU			Antidepressant use at FU			Any NMUPD at FU		
*N* = 5308	no (%)	yes (%)	*p*	no (%)	yes (%)	*p*	no (%)	yes (%)	*p*
Physical assault by family member (ref. no)	4.0	7.9	.070	4.0	9.2	.035	4.0	5.2	.187
Sexual assault by family member (ref. no)	1.1	5.6	< .001	1.2	4.6	.011	1.1	2.6	.003
Physical assault by a stranger (ref. no)	9.5	27.0	< .001	9.5	27.7	< .001	9.1	15.8	< .001
Sexual assault by a stranger (ref. no)	0.9	5.6	< .001	0.9	9.2	< .001	0.8	2.2	.003
Number of potentially traumatic events (ref. none)			< .001			< .001			< .001
one or two	31.6	40.4		31.6	38.5		31.0	38.8	
three or more	12.6	30.3		12.6	36.9		12.2	19.4	
Problems with family within past year (ref. no)			< .001			< .001			< .001
once or twice	16.5	19.1		16.5	21.5		15.8	23.0	
three times or more	6.3	21.3		6.2	32.3		5.9	13.2	
Problems with friends within past year (ref. no)			< .001			< .001			< .001
once or twice	19.9	29.2		19.9	35.4		19.1	29.8	
three times or more	4.2	13.5		4.2	15.4		4.0	8.2	
Unsatisfactory relationship with parents (ref. satisfactory)	13.4	28.1	< .001	13.3	36.9	< .001	13.1	18.2	.002
Lack of parental support (ref. adequate support)	8.8	16.9	.008	8.8	20.0	.002	8.7	11.0	.082
Lack of parental monitoring (ref. adequate monitoring)	18.0	33.7	< .001	18.2	27.7	.048	18.1	20.2	.242
Parents having mental health problems (ref. no)	10.9	22.0	.001	10.9	24.6	< .001	10.6	16.0	< .001

The upper part shows potentially traumatic events, the lower part stressors related to the social environment. FU = follow-up assessment; NMUPD = non-medical use of prescription drugs; ref. = reference.

**Table 3.** Log-binomial regression models of all evaluated variables retained after backward elimination predicting NMUPD outcomes.

	Sleeping pill use at FU			Tranquilizer use at FU			Opioid analgesics use at FU		
		CI 95%			CI 95%			CI 95%	
*N* = 5308	RR	lower limit	upper limit	RR	lower limit	upper limit	RR	lower limit	upper limit
Physical assault by family member (ref. no)									
Sexual assault by family member (ref. no)	2.91**	1.54	5.49						
Physical assault by a stranger (ref. no)							1.46**	1.10	1.94
Sexual assault by a stranger (ref. no)				3.01**	1.48	6.10			
Number of potentially traumatic events (ref. none)									
one or two	1.50*	1.04	2.14	1.18	0.78	1.78	1.57***	1.23	2.01
three or more	1.85**	1.20	2.85	1.81*	1.14	2.87	1.92***	1.42	2.59
Problems with family within past year (ref. no)									
once or twice	1.35	0.90	2.01	1.79**	1.18	2.71	1.20	0.91	1.59
three times or more	2.45***	1.56	3.85	2.05*	1.22	3.42	1.70**	1.21	2.40
Problems with friends within past year (ref. no)									
once or twice				1.56*	1.05	2.33	1.39*	1.08	1.79
three times or more				2.34**	1.37	4.01	1.38	0.90	2.11
Unsatisfactory relationship with parents (ref. satisfactory)	1.49*	1.00	2.20						
Lack of parental support (ref. adequate support)									
Lack of parental monitoring (ref. adequate monitoring)									
Parents having mental health problems (ref. no)				1.70*	1.13	2.56			
	Psychostimulant use at FU			Antidepressant use at FU			Any NMUPD at FU		
		CI 95%			CI 95%			CI 95%	
*N* = 5308	RR	lower limit	upper limit	RR	lower limit	upper limit	RR	lower limit	upper limit
Physical assault by family member (ref. no)									
Sexual assault by family member (ref. no)									
Physical assault by a stranger (ref. no)	2.01***	1.23	3.27				1.31*	1.04	1.65
Sexual assault by a stranger (ref. no)				4.64***	2.07	10.42			
Number of potentially traumatic events (ref. none)									
one or two	2.05**	1.23	3.41	2.42**	1.29	4.51	1.47***	1.22	1.78
three or more	2.93***	1.64	5.22	3.76***	1.92	7.35	1.56***	1.23	1.99
Problems with family within past year (ref. no)									
once or twice	1.24	0.72	2.14	1.83	0.97	3.45	1.37**	1.11	1.70
three times or more	3.08***	1.82	5.21	5.66***	3.23	9.91	1.81***	1.39	2.37
Problems with friends within past year (ref. no)									
once or twice							1.45***	1.19	1.76
three times or more							1.52*	1.10	2.10
Unsatisfactory relationship with parents (ref. satisfactory)									
Lack of parental support (ref. adequate support)									
Lack of parental monitoring (ref. adequate monitoring)	1.72*	1.10	2.68						
Parents having mental health problems (ref. no)									

The upper part shows potentially traumatic events, the lower part stressors related to the social environment. FU = follow-up assessment; NMUPD = non-medical use of prescription drugs; ref. = reference; RR = Risk Ratio; CI = Confidence Interval; *p<.5; **p<.01;***p<.001.

Among social-environmental stressors, only recent, past 12-months problems with the family (RR between 1.70 and 5.66) were consistently significantly associated with NMUPD of all classes, whereas lack of parental support was not associated with any.

Unsatisfactory relationships with parents were only significantly associated with the NMU of sleeping pills (RR = 1.49, *p* < .05). Lack of parental monitoring was only significantly associated with use of stimulants (RR = 1.72, *p* < .05), and having a parent who had suffered from a mental disorder was only significantly associated with the NMU of tranquilizers (RR = 1.70, *p* < .05). Having problems with friends was significantly associated with the NMU of tranquilizers and opioid analgesics.

Finally, the NMU of any prescribed drug was significantly associated with the cumulative number of potentially traumatic events, problems with the family and with friends, and physical assault by a stranger, thus mirroring the associations with the most prevalent drug class, namely opioid analgesics.

## 4. Discussion

Our results show that NMUPD is associated with exposure to both forms of stressors, discrete, potentially traumatic events and chronic social-environmental stressors. For potentially traumatic events this could be consistently shown for the cumulative number of these events and corresponds well with findings in the literature (McCauley et al., 2010). Thus, our findings confirm previous reports that both forms of stressors, discrete traumatic events but also more subtle social-environmental stressors, are important for mental health aspects related to SU (Ames & Roitzsch, 2000).

The link between traumatic stressors and NMUPD is particularly well established for a diagnosis of PTSD and the NMU of opioid medications (Smith et al., 2016). In the present study, the most malicious forms of interpersonal violence – physical or sexual assault – were not only associated with opioid medication but also to various degrees with all outcomes of NMUPD. However, more significant associations were found with assaults by strangers. Against our hypothesis, there was only one significant relationship for assaults within the family, which was associated with NMU of sleeping pills. Given that many occurrences of sexual abuse by a family member occur at night or in bed, it is possible that this type of drug is more likely to be used by men who have experienced this sort of trauma, perhaps as a form of self-medicating to induce sleep or to avoid the emotions associated with bedtime traumatic reminders. Further research would be useful to further clarify this association.

Fewer significant associations for assaults by family members than by strangers might be explained by the fact that the sample only investigated young men. Indeed, it has been shown that traumatic events have different effects on subsequent SU when male and female samples are compared (Simpson & Miller, 2002). For instance, a lifetime history of sexual assault was significantly correlated with alcohol abuse in females, but not in males, whereas a lifetime history of physical assault or a comorbid diagnosis of PTSD was significantly associated with alcohol abuse in men, but not in women (Danielson et al., 2009). In female samples, it was shown that a history of PTSD and of a drug-alcohol facilitated rape highly predicted NMUPD (McCauley et al., 2009), and that a history of sexual violence was specifically associated with an increased use of opioid medication and sedatives (Young, Grey, Boyd, & McCabe, 2011). There is little research for specific effects of assaults on men. However, Smith et al. (2016) showed that a diagnosis of PTSD is more strongly associated with NMU of opioid medication in women in comparison to men. Further, when comparing male with female samples that have been sexually assaulted during childhood and youth it was shown that odds ratios for abuse of alcohol or of illicit drugs was higher in females than in males (Dube et al., 2005).

Thus, even though men seem to react differently to interpersonal violence than women, it is somehow striking that sexual assaults perpetrated within the family showed only one significant association with the tested NMUPD groups. However, it is important to remember that in bivariate analysis, sexual assault within family was associated with all NMUPD outcomes. It is possible that in multiple regressions other family-related variables (e.g. problems within family), which are related to a conflict-loaded, disruptive atmosphere within families, have shared variance with assaults, rendering the effect of assaults non-significant in multiple models. Our findings concerning the lack of association between sexual and physical assaults by family members and NMUPD in young men can only be generalized with caution as our analysis was based on relatively low numbers of occurrences resulting in low statistical power in comparison to the other investigated stressors. With regard to the finding that assaults perpetrated by strangers showed more frequently associations with NMUPD than assaults within the family it is possible that feelings of guilt and shame which contribute to higher rates of SU in men (Rahim & Patton, 2015) are more pronounced in young men when the assault takes place in a more visible social context than in discrete and hidden family settings.

Our results further showed that stress resulting from problems with friends or families contributes substantially to NMUPD. These findings are in line with studies indicating that adolescents with strong bonds to their family or school are less likely to engage in NMUPD (Cerda et al., 2014; Ford, 2009). Intense conflicts with parents have been shown to be significantly correlated with the NMU of psychostimulants by adolescents (Herman-Stahl et al., 2006). As to the influence of their relationships with peers, the literature indicates that deviant behaviour by peers, including NMUPD or the use of illicit substances, contributes highly to the transgression of norms, NMUPD, and SU in adolescents (Beal, Ausiello, & Perrin, 2001; Loke, Mak, & Wu, 2016). However, little is known about how conflicts with peers affect SU or NMUPD. A positive relationship between SU and having been a victim of bullying during adolescence has been reported (Radliff, Wheaton, Robinson, & Morris, 2012; Young, Glover, & Havens, 2012); to date, no studies have investigated the links between social stress among adolescents or young adults and NMUPD. Our results suggest that the presence of a conflict-loaded environment between adolescents and their peers might be a risk factor for NMUPD.

Parental monitoring of an adolescent’s choice of peers has been shown to be an important protective factor of SU (Van Ryzin, Fosco, & Dishion, 2012). Several studies have also established the important protective effects of parental involvement and monitoring on adolescent NMUPD (Sung et al., 2005; Vaughn, Fu, Perron, & Wu, 2012). When examining the relationship between parental monitoring and support with regard to the use of alcohol and illicit substances, a recent study on the same C-SURF sample revealed a highly consistent association between a lack of parental monitoring and subsequent use of alcohol and drugs (Rougemont-Bücking, Grazioli, Daeppen, Gmel, & Studer, 2017). In this light, the present results, showing only one significant association between NMUPD and the lack of parental monitoring, are difficult to interpret. It may be that parental monitoring has other effects on NMUPD compared with SU. It has also been shown that increased parental support can be a consequence of the parents’ awareness of their children’s increased use of alcohol (Reimuller, Shadur, & Hussong, 2011). Therefore, parental support and monitoring can be the result of psychological suffering or self-medication with NMPDU in adolescents, thus counterbalancing the effects of lack of monitoring and support as risk factor for NMPDU. Further, there was a consistent association between all drug classes and recent problems with the family. A possible explanation is that problems in the family were already present in childhood and adolescence as indicated in bivariate associations, and that they persisted into young adulthood. Thus, recent problems with family may share variance with childhood and adolescent stressors (such as parental monitoring), rendering their association non-significant in multiple regressions.

Similar difficulties apply to the finding that the presence of a mental disorder in one’s parents was not significantly associated with NMUPD outcomes, with the exception of tranquilizer use. Stressors such as parents being treated for chronic pain or having their own problems with NMUPD or SU are well established risk factors for the initiation of NMUPD in adolescents (Boyd et al., 2006; Nargiso, Ballard, & Skeer, 2015; Tucker et al., 2015). Thus, when assessing the influence of this family factor on NMUPD, one would have expected stronger associations. As most studies have focused on the relational or educational attitudes of parents, there is dearth in findings concerning the effects of parents’ mental health problems on their children’s NMUPD. These potential links deserve further research.

One limitation of this study is the lack of measurements of the severity for most of the stressors, which makes it impossible to weight the specific impact of the independent variables. There was also no information available about whether the various traumatic events had a clinically relevant outcome, such as PTSD. Thus, the available data did not allow distinguishing between stressful, potentially traumatic, and manifestly traumatic events. In addition, since the reference period for stressful events preceded the reference period of NMUPD, it is adequate to interpret results in the direction that stressful events may predispose individuals to use such substances. However, as we were not able to determine the age at which the event occurred one cannot rule out the possibility that the findings may be partially attributable to a reverse causation, i.e. early NMUPD may contribute to being exposed to stress. Finally, another limitation applies to the fact that some responses might be biased by the fear that confidentiality might not be fully guaranteed as this study’s recruitment was organized at the beginning in collaboration with the military enrolment. There also might be underreporting of sexual assaults within families as this is highly intimate and shameful information which some study participants might not be willing to reveal.

In conclusion, this study showed that NMUPD by young men was greatly influenced by prior exposure to one or more potentially traumatic events. Recent problems with family or friends were other stressors highly associated with NMUPD. Interestingly, stressors describing inherent family functioning showed very few associations with NMUPD.

## References

[CIT0001] Ali M. M., Dean D. Jr., Lipari R., Dowd W. N., Aldridge A. P., & Novak S. P. (2015). The mental health consequences of nonmedical prescription drug use among adolescents. *Journal of Mental Health Policy & Economics*, 18(1), 3–12. 25862204

[CIT0002] Ames S. C., & Roitzsch J. C. (2000). The impact of minor stressful life events and social support on cravings: A study of inpatients receiving treatment for substance dependence. *Addictive Behaviors*, 25(4), 539–547. 10972445 10.1016/s0306-4603(00)00069-1

[CIT0003] Baggio S., Studer J., Mohler-Kuo M., Daeppen J. B., & Gmel G. (2014). Non-medical prescription drug and illicit street drug use among young Swiss men and associated mental health issues. *International Journal of Adolescent Medecine & Health*, 26(4), 525–530. 10.1515/ijamh-2013-0330 24447983

[CIT0004] Barrett S. P., Meisner J. R., & Stewart S. H. (2008). What constitutes prescription drug misuse? Problems and pitfalls of current conceptualizations. *Current Drug Abuse Reviews*, 1(3), 255–262. 19630724 10.2174/1874473710801030255

[CIT0005] Beal A. C., Ausiello J., & Perrin J. M. (2001). Social influences on health-risk behaviors among minority middle school students. *Journal of Adolescent Health*, 28(6), 474–480. 11377991 10.1016/s1054-139x(01)00194-x

[CIT0006] Booth J. M., & Anthony E. K. (2015). Examining the interaction of daily hassles across ecological domains on substance use and delinquency among low-income adolescents of color. *Journal of Human Behavior in the Social Environment*, 25(8), 810–821.

[CIT0007] Boyd C. J., Esteban McCabe S., & Teter C. J. (2006). Medical and nonmedical use of prescription pain medication by youth in a Detroit-area public school district. *Drug and Alcohol Dependency*, 81(1), 37–45. 10.1016/j.drugalcdep.2005.05.017 PMC1785363 16040201

[CIT0008] Boyd C. J., McCabe S. E., Cranford J. A., & Young A. (2006). Adolescents’ motivations to abuse prescription medications. *Pediatrics*, 118(6), 2472–2480. 17142533 10.1542/peds.2006-1644 PMC1785364

[CIT0009] Brands B., Paglia-Boak A., Sproule B. A., Leslie K., & Adlaf E. M. (2010). Nonmedical use of opioid analgesics among Ontario students. *Canadian Family Physician*, 56(3), 256–262. 20228312 PMC2837694

[CIT0010] Bureau J.-F., Jodi M., & Lyons-Ruth K. (2010). Attachment dysregulation as hidden trauma in infancy: Early stress, maternal buffering and psychiatric morbidity in young adulthood In Lanius R., Vermetten E., & Pain C. (Eds.), *The impact of early life trauma on health and disease: The hidden epidemic* (pp. 48–56). New York: Cambridge University Press.

[CIT0011] Cerda M., Bordelois P., Keyes K. M., Roberts A. L., Martins S. S., Reisner S. L., … Koenen K. C. (2014). Family ties: Maternal-offspring attachment and young adult nonmedical prescription opioid use. *Drug and Alcohol Dependency*, 142, 231–238. 10.1016/j.drugalcdep.2014.06.026 PMC4134317 25024105

[CIT0012] Chamberlain K., & Zika S. (1990). The minor events approach to stress: Support for the use of daily hassles. *British Journal of Psychology*, 81(Pt 4), 469–481. 2279232 10.1111/j.2044-8295.1990.tb02373.x

[CIT0013] Charuvastra A., & Cloitre M. (2008). Social bonds and posttraumatic stress disorder. *Annual Review of Psychology*, 59, 301–328. 10.1146/annurev.psych.58.110405.085650 PMC2722782 17883334

[CIT0014] Conn B. M., & Marks A. K. (2014). Ethnic/Racial differences in peer and parent influence on adolescent prescription drug misuse. *Journal of Developmental and Behavioral Pediatrics*, 35(4), 257–265. 24799264 10.1097/DBP.0000000000000058

[CIT0015] C-SURF 2017 Retrieved 112, 2017 from http://www.c-surf.ch/img/questionnaires_pdf/q2_follow_up_en.pdf

[CIT0016] D’Amico E. J., & McCarthy D. M. (2006). Escalation and initiation of younger adolescents’ substance use: The impact of perceived peer use. *Journal of Adolescent Health*, 39(4), 481–487. 16982381 10.1016/j.jadohealth.2006.02.010

[CIT0017] Danielson C. K., Amstadter A. B., Dangelmaier R. E., Resnick H. S., Saunders B. E., & Kilpatrick D. G. (2009). Trauma-related risk factors for substance abuse among male versus female young adults. *Addictive Behaviors*, 34(4), 395–399. 19110381 10.1016/j.addbeh.2008.11.009 PMC2704020

[CIT0018] Dube S. R., Anda R. F., Whitfield C. L., Brown D. W., Felitti V. J., Dong M., & Giles W. H. (2005). Long-term consequences of childhood sexual abuse by gender of victim. *American Journal of Preventive Medicine*, 28(5), 430–438. 15894146 10.1016/j.amepre.2005.01.015

[CIT0019] Eisenberger N. I. (2012). The neural bases of social pain: Evidence for shared representations with physical pain. *Psychosomatic Medicine*, 74(2), 126–135. 22286852 10.1097/PSY.0b013e3182464dd1 PMC3273616

[CIT0020] Foa E. B., Cashman L., Jaycox L., & Perry K. (1997). The validation of a self-report measure of posttraumatic stress disorder: The posttraumatic diagnostic scale. *Psychological Assessment*, 9(4), 445–451.

[CIT0021] Ford J. A. (2008a). Nonmedical prescription drug use among college students: A comparison between athletes and nonathletes. *Journal of American College Health*, 57(2), 211–219. 18809538 10.3200/JACH.57.2.211-220

[CIT0022] Ford J. A. (2008b). Social learning theory and nonmedical prescription drug use among adolescents. *Sociological Spectrum*, 28(3), 299–316.

[CIT0023] Ford J. A. (2009). Nonmedical prescription drug use among adolescents the influence of bonds to family and school. *Youth & Society*, 40(3), 336–352.

[CIT0024] Ford J. A., & Arrastia M. C. (2008). Pill-poppers and dopers: A comparison of non-medical prescription drug use and illicit/street drug use among college students. *Addictive Behaviors*, 33(7), 934–941. 18403131 10.1016/j.addbeh.2008.02.016

[CIT0025] Ford J. A., Reckdenwald A., & Marquardt B. (2014). Prescription drug misuse and gender. *Substance Use and Misuse*, 49(7), 842–851. 24491149 10.3109/10826084.2014.880723

[CIT0026] Gmel G., Akre C., Astudillo M., Bähler C., Baggio S., Bertholet N., … Deline S. (2015). The Swiss cohort study on substance use risk factors–Findings of two waves. *Sucht*, 61(4), 251–262.

[CIT0027] Gray M. J., Litz B. T., Hsu J. L., & Lombardo T. W. (2004). Psychometric properties of the life events checklist. *Assessment*, 11(4), 330–341. 15486169 10.1177/1073191104269954

[CIT0028] Herman-Stahl M. A., Krebs C. P., Kroutil L. A., & Heller D. C. (2006). Risk and protective factors for nonmedical use of prescription stimulants and methamphetamine among adolescents. *Journal of Adolescent Health*, 39(3), 374–380. 16919799 10.1016/j.jadohealth.2006.01.006

[CIT0029] Hibell B., Guttormsson U., Ahlström S., Balakireva O., Bjarnason T., Kokkevi A., & Kraus L. (2012). *The 2011 ESPAD report. Substance use among students in 36 European Countries*. Tukholma: The Swedish Council for Information on Alcohol and other Drugs, 2012 Viitattu 27.9. 2013.

[CIT0030] Hooper L. M., Stockton P., Krupnick J. L., & Green B. L. (2011). Development, use, and psychometric properties of the trauma history questionnaire. *Journal of Loss & Trauma*, 16(3), 258–283.

[CIT0031] Joffe A. (2014). Nonmedical use of prescription stimulants by adolescents. *Adolescent Medicine: State of the Art Reviews*, 25(1), 89–103. 25022188

[CIT0032] Johnston L. D. (2009). Prescription drug use by adolescents: What we are learning and what we still need to know. *Journal of Adolescent Health*, 45(6), 539–540. 19931823 10.1016/j.jadohealth.2009.09.004

[CIT0033] Kelly B. C., Wells B. E., Leclair A., Tracy D., Parsons J. T., & Golub S. A. (2013). Prevalence and correlates of prescription drug misuse among socially active young adults. *International Journal on Drug Policy*, 24(4), 297–303. 23036649 10.1016/j.drugpo.2012.09.002 PMC3546267

[CIT0034] Kelly B. C., Wells B. E., Pawson M., LeClair A., & Parsons J. T. (2014). Combinations of prescription drug misuse and illicit drugs among young adults. *Addictive Behaviors*, 39(5), 941–944. 24462348 10.1016/j.addbeh.2013.12.003 PMC3980000

[CIT0035] Loke A. Y., Mak Y. W., & Wu C. S. (2016). The association of peer pressure and peer affiliation with the health risk behaviors of secondary school students in Hong Kong. *Public Health*, 137, 113–123. 27062065 10.1016/j.puhe.2016.02.024

[CIT0036] Lowe S. R., Sampson L., Young M. N., & Galea S. (2017). Alcohol and nonmedical prescription drug use to cope with posttraumatic stress disorder symptoms: An analysis of hurricane sandy survivors. *Substance Use and Misuse*, 52(10), 1348–1356. 28394737 10.1080/10826084.2017.1280832

[CIT0037] McCabe S. E., Kloska D. D., Veliz P., Jager J., & Schulenberg J. E. (2016). Developmental course of non-medical use of prescription drugs from adolescence to adulthood in the USA: National longitudinal data. *Addiction*, 111(12), 2166–2176. 27338559 10.1111/add.13504 PMC5183528

[CIT0038] McCabe S. E., West B. T., & Boyd C. J. (2013). Motives for medical misuse of prescription opioids among adolescents. *Journal of Pain*, 14(10), 1208–1216. 23954519 10.1016/j.jpain.2013.05.004 PMC3792708

[CIT0039] McCauley J. L., Amstadter A. B., Danielson C. K., Ruggiero K. J., Kilpatrick D. G., & Resnick H. S. (2009). Mental health and rape history in relation to non-medical use of prescription drugs in a national sample of women. *Addictive Behaviors*, 34(8), 641–648. 19375238 10.1016/j.addbeh.2009.03.026 PMC2738927

[CIT0040] McCauley J. L., Danielson C. K., Amstadter A. B., Ruggiero K. J., Resnick H. S., Hanson R. F., … Kilpatrick D. G. (2010). The role of traumatic event history in non-medical use of prescription drugs among a nationally representative sample of US adolescents. *Journal of Child Psychology and Psychiatry and Allied Disciplines*, 51(1), 84–93. 10.1111/j.1469-7610.2009.02134.x PMC2826795 19674194

[CIT0041] McIntyre K. P., Korn J. H., & Matsuo H. (2008). Sweating the small stuff: How different types of hassles result in the experience of stress. *Stress and Health*, 24(5), 383–392.

[CIT0042] McLellan A. T., Luborsky L., Woody G. E., & O’Brien C. P. (1980). An improved diagnostic evaluation instrument for substance abuse patients. The addiction severity index. *Journal of Nervous and Mental Disease*, 168(1), 26–33. 7351540 10.1097/00005053-198001000-00006

[CIT0043] N’Goran A. A., Baggio S., Deline S., Studer J., Mohler-Kuo M., Daeppen J. B., & Gmel G. (2015). Association between non-medical prescription drug use and personality traits among young Swiss men. *Psychiatry and Clinical Neurosciences*, 69(4), 228–237. 25113854 10.1111/pcn.12231

[CIT0044] Nargiso J. E., Ballard E. L., & Skeer M. R. (2015). A systematic review of risk and protective factors associated with nonmedical use of prescription drugs among youth in the USA: A social ecological perspective. *Journal of Studies on Alcohol & Drugs*, 76(1), 5–20. 25486389

[CIT0045] O’brien R. M. (2007). A caution regarding rules of thumb for variance inflation factors. *Quality & Quantity*, 41(5), 673–690.

[CIT0046] Pedersen C. A. (2004). Biological aspects of social bonding and the roots of human violence. *Annals of the New York Academy of Sciences*, 1036(1), 106–127. 15817733 10.1196/annals.1330.006

[CIT0047] Peralta R. L., & Steele J. L. (2010). Nonmedical prescription drug use among US college students at a Midwest university: A partial test of social learning theory. *Substance Use and Misuse*, 45(6), 865–887. 20397873 10.3109/10826080903443610

[CIT0048] Peralta R. L., Stewart B. C., Steele J. L., & Wagner F. A. (2016). Nonmedical use of prescription drugs in emerging adulthood: Differentiating sex from gender. *Addiction Research & Theory*, 24(5), 389–397. 28090200 10.3109/16066359.2016.1140745 PMC5223279

[CIT0049] Pinch W. J., Heck M., & Vinal D. (1986). Health needs and concerns of male adolescents. *Adolescence*, 21(84), 961–969. 3825675

[CIT0050] Quinn K., Boone L., Scheidell J. D., Mateu-Gelabert P., McGorray S. P., Beharie N., … Khan M. R. (2016). The relationships of childhood trauma and adulthood prescription pain reliever misuse and injection drug use. *Drug and Alcohol Dependence*, 169, 190–198. 27816251 10.1016/j.drugalcdep.2016.09.021 PMC5728665

[CIT0051] Radliff K. M., Wheaton J. E., Robinson K., & Morris J. (2012). Illuminating the relationship between bullying and substance use among middle and high school youth. *Addictive Behaviors*, 37(4), 569–572. 22277772 10.1016/j.addbeh.2012.01.001

[CIT0052] Rahim M., & Patton R. (2015). The association between shame and substance use in young people: A systematic review. *Peer Journal*, 3(p), e737. 10.7717/peerj.737 PMC4312064 25649509

[CIT0053] Reimuller A., Shadur J., & Hussong A. M. (2011). Parental social support as a moderator of self-medication in adolescents. *Addictive Behaviors*, 36(3), 203–208. 21146938 10.1016/j.addbeh.2010.10.006 PMC3023817

[CIT0054] Rigg K. K., & Ibanez G. E. (2010). Motivations for non-medical prescription drug use: A mixed methods analysis. *Journal of Substance Abuse Treatment*, 39(3), 236–247. 20667680 10.1016/j.jsat.2010.06.004 PMC2937068

[CIT0055] Rougemont-Bücking A., Grazioli V. S., Daeppen J. B., Gmel C., & Studer J. (2017). Family-related stress versus external stressors: Differential impacts on alcohol and illicit drug use in young men. *European Addiction Research*, 23(6), 284–297. 29275419 10.1159/000485031

[CIT0056] Simoni-Wastila L., Ritter G., & Strickler G. (2004). Gender and other factors associated with the nonmedical use of abusable prescription drugs. *Substance Use and Misuse*, 39(1), 1–23. 15002942 10.1081/ja-120027764

[CIT0057] Simpson T. L., & Miller W. R. (2002). Concomitance between childhood sexual and physical abuse and substance use problems. A review. *Clinical Psychology Review*, 22(1), 27–77. 11793578 10.1016/s0272-7358(00)00088-x

[CIT0058] Sinha R. (2008). Chronic stress, drug use, and vulnerability to addiction. *Annals of the New York Academy of Sciences*, 1141, 105–130. 18991954 10.1196/annals.1441.030 PMC2732004

[CIT0059] Smith K. Z., Smith P. H., Cercone S. A., McKee S. A., & Homish G. G. (2016). Past year non-medical opioid use and abuse and PTSD diagnosis: Interactions with sex and associations with symptom clusters. *Addictive Behaviors*, 58, 167–174. 26946448 10.1016/j.addbeh.2016.02.019 PMC4808454

[CIT0060] Studer J., Baggio S., Mohler-Kuo M., Dermota P., Gaume J., Bertholet N., … Gmel G. (2013). Examining non-response bias in substance use research—Are late respondents proxies for non-respondents? *Drug and Alcohol Dependence*, 132(1), 316–323. 23535061 10.1016/j.drugalcdep.2013.02.029

[CIT0061] Sung H. E., Richter L., Vaughan R., Johnson P. B., & Thom B. (2005). Nonmedical use of prescription opioids among teenagers in the USA: Trends and correlates. *Journal of Adolescent Health*, 37(1), 44–51. 15963906 10.1016/j.jadohealth.2005.02.013

[CIT0062] Taeger D., Sun Y., & Straif K. (1998). On the use, misuse and interpretation of odds ratios. *British Medical Journal*, 316(7136), 989.9550961

[CIT0063] Tucker J. S., Ewing B. A., Miles J. N., Shih R. A., Pedersen E. R., & D’Amico E. J. (2015). Predictors and consequences of prescription drug misuse during middle school. *Drug and Alcohol Dependence*, 156, 254–260. 26455553 10.1016/j.drugalcdep.2015.09.018 PMC4640892

[CIT0064] Van Ryzin M. J., Fosco G. M., & Dishion T. J. (2012). Family and peer predictors of substance use from early adolescence to early adulthood: An 11-year prospective analysis. *Addictive Behaviors*, 37(12), 1314–1324. 22958864 10.1016/j.addbeh.2012.06.020 PMC3459354

[CIT0065] Vaughn M. G., Fu Q., Perron B. E., & Wu L. T. (2012). Risk profiles among adolescent nonmedical opioid users in the USA. *Addictive Behaviors*, 37(8), 974–977. 22525786 10.1016/j.addbeh.2012.03.015 PMC3358501

[CIT0066] Wigman J. T., van Os J., Thiery E., Derom C., Collip D., Jacobs N., & Wichers M. (2013). Psychiatric diagnosis revisited: Towards a system of staging and profiling combining nomothetic and idiographic parameters of momentary mental states. *PloS One*, 8(3), e59559. 23555706 10.1371/journal.pone.0059559 PMC3610753

[CIT0067] Williamson T., Eliasziw M., & Fick G. H. (2013). Log-binomial models: Exploring failed convergence. *Emerging Themes in Epidemiology*, 10(1), 14. 24330636 10.1186/1742-7622-10-14 PMC3909339

[CIT0068] Young A., Grey M., Boyd C. J., & McCabe S. E. (2011). Adolescent sexual assault and the medical and nonmedical use of prescription medication. *Journal of Addictions Nursing*, 11(1–2), 25–31. 22065397 10.3109/10884601003628138 PMC3208183

[CIT0069] Young A. M., Glover N., & Havens J. R. (2012). Nonmedical use of prescription medications among adolescents in the USA: A systematic review. *Journal of Adolescent Health*, 51(1), 6–17. 22727071 10.1016/j.jadohealth.2012.01.011

[CIT0070] Zellner M. R., Watt D. F., Solms M., & Panksepp J. (2011). Affective neuroscientific and neuropsychoanalytic approaches to two intractable psychiatric problems: Why depression feels so bad and what addicts really want. *Neuroscience and Biobehavioral Reviews*, 35(9), 2000–2008. 21241736 10.1016/j.neubiorev.2011.01.003

